# Could the Evaluation of Muscle Strength Imbalances Be Used as a Predictor of Total Hip Arthroplasty?

**DOI:** 10.3390/ijerph18105082

**Published:** 2021-05-11

**Authors:** Tomáš Vodička, Michal Bozděch, Marta Gimunová, Lenka Svobodová, Jiří Zháněl, Stanisław Henryk Czyż

**Affiliations:** 1 Faculty of Sport Studies, Masaryk University, 60177 Brno, Czech Republic; michal.bozdech@fsps.muni.cz (M.B.); gimunova@fsps.muni.cz (M.G.); netyaloty@post.cz (L.S.); 116498@mail.muni.cz (J.Z.); stanislaw.czyz@fsps.muni.cz (S.H.C.); 2 Faculty of Sport and Physical Education, University School of Physical Education in Wrocław, 51-612 Wrocław, Poland; 3 Physical Activity, Sport and Recreation Focus Group, North-West University, Potchefstroom 2520, South Africa

**Keywords:** osteoarthritis, isokinetic dynamometry, total hip arthroplasty, muscular imbalances

## Abstract

Loss of muscle strength characterizes the period before total hip arthroplasty (THA). Little is known about whether muscle strength imbalances caused by muscle strength decline could be considered another clinical predictor for THA. This study aimed to determine whether muscle imbalances may be used as a clinical predictor for THA surgery. Thirty-six participants were enrolled in the study. Eighteen patients had THA (THA group), while 18 were healthy elders (CON group). Ipsilateral (H/Q) and bilateral (% Def) muscle imbalances of the knee were assessed. THA patients showed impairment of the extensors on the affected extremity compared to those unaffected. A comparison between the groups proved there were weakened flexors in the THA group on both extremities. A comparison of the imbalances revealed a significant bilateral imbalance of the extensors and ipsilateral imbalance of both extremities in the THA group. We computed two logistic regressions using bilateral and ipsilateral imbalance as the predictors of THA surgery. We found that bilateral extensor imbalance may be used as a predictor for THA (Nagelkerke R^2^ = 0.22). A decrease of the bilateral extensors imbalance by 8% decreases the probability of THA by 8%. The most interesting finding is that the evaluation of the bilateral extensor imbalance may be used as another clinical predictor for THA.

## 1. Introduction

Osteoarthritis (OA) is the most prevalent chronic rheumatic disease worldwide, with a strong impact on individual and population health [1], and its prevalence is estimated to increase as the population ages. The reports from the World Health Organization indicate that approximately 10% of men and 18% of women aged 60 years or over have symptomatic OA [2].

OA is characterized by pain, a reduction of physical function, decreased range of motion, muscle weakness and joint instability [3]. These conditions lead to a reduced quality of life, with a decline in the ability to perform daily activities, especially in older adults [4]. The knee and hip are frequently affected by OA, because they are the joints most involved in weight-bearing during human locomotion [5].

Radiographic screening (X-ray) is the most widely used screening method for hip OA [6,7], especially in moderate-to-severe hip OA, but may be asymptomatic [6]. Magnetic Resonance Imaging (MRI) is more useful than radiography in detecting early structural changes such as focal cartilage defects and bone marrow lesions in the subchondral bone [3]. It is, however, the most expensive method. Hip OA can also be diagnosed by clinical criteria—a combination of history and physical examination findings without the need to expose the patient to unnecessary radiation [3].

In patients with progressive hip OA was revealed a generalized muscle weakness of the affected extremity [8,9], reduced muscle density [10] and muscle inhibition [11]. Muscle weakness and muscle imbalances identified by diagnostic screening are not considered a clinical symptom of hip OA, in spite of the fact that the muscle weakness could be a predisposing factor for the development and progression of hip OA [3,8]. This is quite surprising, since the period before total hip arthroplasty (THA), the final treatment of hip OA, is characterized by a radical loss of muscle strength in the lower extremities [12].

Previous research has focused mostly on the changes in muscle strength in the postoperative period [9,13,14,15,16,17]. However, little is known about muscle strength imbalances in patients facing THA in the preoperative period and whether muscle imbalances could be used for clinical screening. From a practical point of view, if muscle imbalances could serve as predictors for THA, then this could be assessed during symptom screening without or before sending the patient for an X-ray or MRI. It could also be used as an OA progress indicator instead of expensive and nonhealthy radiological assessments.

We formulated two objectives assuming the facts mentioned above. The primary purpose was to compare the knee joint’s muscle imbalances in the affected and unaffected lower extremities in the preoperative period of patients indicated for THA due to hip OA. The secondary objective was to assess whether muscle imbalances may be used as predictors for THA surgery, i.e., as another clinical indicator tested during symptom screening.

## 2. Materials and Methods

### 2.1. Participants

The sample size was calculated based on the means and standard deviations presented by Fukumoto et al. [15]. We used values reported for the extensors and flexors of the affected and unaffected lower extremities in the THA patients and the stronger and weaker lower extremities in the healthy elderly participants (control group, CON) reported in the study by Fukumoto et al. [15]. The confidence interval was set at 95% and the power at 0.8. The sample size was calculated using Open Epi software, version 2 (Open Source Epidemiologic Statistics for Public Health; www.OpenEpi.com (accessed on 1 February 2021)) [18]. The calculated sample size was 10 participants in total. Nevertheless, we decided to recruit a larger number to prevent problems with potential dropout. THA male patients (*n* = 18; age: 58.38 ± 5.30 years; height: 178.67 ± 6.58 cm; weight: 99.41 ± 17.81 kg) were conveniently selected from the database of the Orthopaedic Department at St Anne’s University Hospital in Brno, Czech Republic. These patients underwent unilateral primary THA due to hip OA using an anterolateral surgical approach based on Watson-Jones and were able to ambulate without assistive devices. The patient severities of OA were classified as grades 3 and 4 based on the Kellgren-Lawrence classification of osteoarthritis. All patients have one-sided primary degenerative changes without aseptic necrosis of the femoral head. Eventually, 18 elderly THA patients met the criteria and volunteered to participate in the study.

Healthy elderly male adults with no hip OA diagnosis were recruited to the matched CON group (*n* = 18; age: 64.64 ± 3.57 years; height 175.81 ± 7.41 cm; weight 95.54 ± 14.58 kg). They were conveniently selected from the University of the Third Age at Masaryk University, Brno.

The exclusion criteria for both groups included: previous orthopaedic surgery in the lower extremity or lumbar spine, lumbar spine pain, lumbar spine degenerative changes, previous knee pathology, a history of neurological diseases, history of the lower extremity or back surgery, previously diagnosed rheumatoid arthritis, formerly diagnosed central or peripheral nervous system involvement and dementia involving decreased cognitive function. The Ethics Committee of St. Anne’s University Hospital in Brno (Czech Republic) approved the study. All participants were informed about the study’s aims and procedures. Informed consent was approved and signed by each participant before participating in the study.

### 2.2. Equipment

A Humac Norm isokinetic dynamometer (Humac Norm, Computer Sports Medicine, Inc., Stoughton, MA, USA) was used to assess the extensor and flexor strengths of the knee joint. Reliability of the use of the Humac Norm dynamometer for knee strength measurements has been proven recently [19].

### 2.3. Procedures

**Figure 1:**
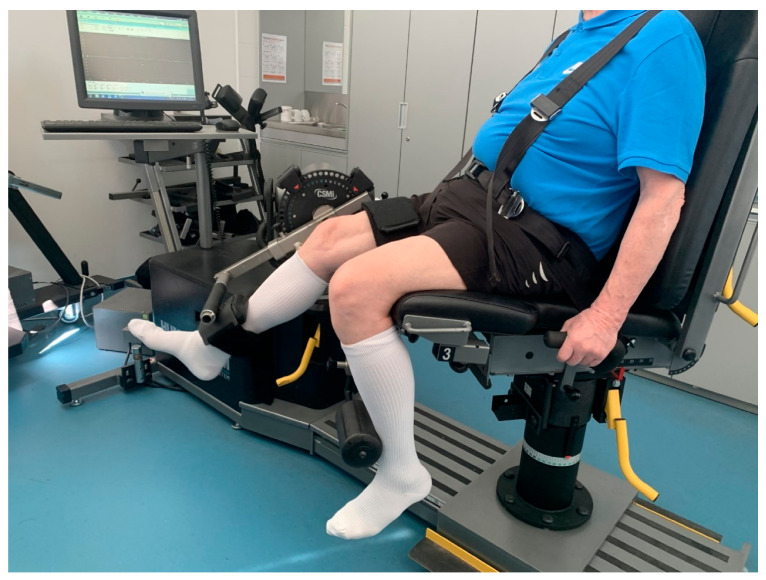
Assessment of isokinetic knee strength testing.

After identifying the THA patients through an electronic database, the investigator contacted the given persons by telephone and provided them with details about the project. Patients willing to participate and satisfying the inclusion criteria were selected. Before the muscular performance measurements, the participants performed five minutes of warm-up (0.5 W/kg) on a Lode bicycle ergometer (Lode Excalibur Sport, Groningen, The Netherlands). After the ergometer warm-up, participants performed five nonmaximal familiarization trials on the dynamometer for each movement. After thirty seconds of pause, the concentric isokinetic knee flexion and extension movements were assessed at an angular velocity of 60°/s (five maximal repetitions) over a range of motion of 90°, from 0° to 90° of knee flexion. The maximal knee extensor and flexor strengths were evaluated as the peak torque (Nm) during isokinetic concentric contraction. The THA group’s unaffected lower extremity was tested first. In the CON group, the test was initiated with a randomly selected lower extremity. The participants sat in the isokinetic dynamometer chair with the back support set at 85°. The pad of the dynamometer was placed superior (3 cm) to the lateral malleolus. The knee joint axis was aligned with the mechanical axis of the dynamometer (Figure 1). During the tests, standardized verbal encouragement was given to ensure maximal force. The participants were not allowed to see the screen during testing. Gravity correction was obtained before each test. Calibration of the isokinetic dynamometer was performed according to the specifications given in the manufacturer’s manual.

### 2.4. Statistical Analysis

We used descriptive statistics to address the first objective. The values were expressed as the mean, standard deviation, ranges and proportions. Our data were not normally distributed (*p* > 0.05 in the Shapiro–Wilk test), except for the variable age in the CON group (*p* < 0.05). Therefore, the differences between the groups and lower extremities were tested with the Mann–Whitney *U* test. The effect size was assessed using Cohen’s *d*, which can be interpreted as small (*d* = 0.20), medium (*d* = 0.50) or large (*d* = 0.80).

We computed two binary logit regression models to address the second objective. In both of these, the dependent variable was whether a lower extremity was affected (coded 1) or unaffected (coded as 0). We used data from both groups (CON and THA). Two predictors were used in the first model: the bilateral strength imbalance between the extensors and the imbalance between the flexors. The predictor used in the second model was the ipsilateral strength imbalance between extensors and flexors in the affected lower extremity (coded as 1) and unaffected lower extremity, in both the THA and CON groups. We used Nagelkerke R^2^ as a measure of the model fit. Standard errors (SE), odds ratio (OR) and 95% CI are reported. We created a Receiver Operator Characteristic (ROC) curve for the statistically significant logistic regressions to show the diagnostic ability of binary classifiers.

## 3. Results

Table 1 shows the participant characteristics in both the THA and CON groups. A significant age difference was found, but there was no significant differences in height and weight between the two groups.

Table 2 assesses the bilateral strength imbalances between the affected and unaffected lower extremities in the THA group and the stronger and weaker extremities in the CON group.

The evaluation of the bilateral strength imbalance of the knee extensors in the THA group revealed a significant muscle strength deficit in the affected extremity (*p* = 0.013, Cohen’s *d* = 0.90). The knee flexors’ bilateral strength imbalance was proven in the CON group (*p* = 0.017; Cohen’s *d* = 0.51).

Table 3 summarizes the results of the muscle strength of the knee extensors and flexors in the THA and CON groups.

Muscle strength imbalance evaluation between the groups (THA and CON) proved that the flexors weakened in both the unaffected (*p* = 0.018, Cohen’s *d* = 0.83) and affected (*p* = 0.028, Cohen’s *d* = 0.71) lower extremities in the THA group. Table 4 describes the evaluation of the bilateral and ipsilateral strength imbalances between the lower extremities and the groups, respectively.

The evaluation of bilateral strength imbalances between the groups (THA and CON) revealed a significant weakening of knee extensors in the THA group (*p* = 0.029, Cohen’s *d* = 0.82): % Def = 18.51%. This finding points to the weakening of the knee extensors of the affected lower extremities in the THA group. A comparison of the ipsilateral imbalances between the groups showed a significantly lower ipsilateral strength imbalance in the THA group in the affected lower extremity (*p* = 0.034, Cohen’s *d* = 0.72): H/Q = 47.69% and in the unaffected lower extremity (*p* < 0.001, Cohen’s *d* = 1.75): H/Q = 42.04%.

### Logistic Regression Models

We computed two logistic models in order to determine if muscle imbalances may be used as predictors of whether a person should undergo THA. Two predictors were used in the first model. The first was the bilateral strength imbalance (in percent) between the flexors in the unaffected and affected (THA group) and stronger and weaker (CON group) lower extremities. Analogically, the second predictor was calculated for a strength imbalance between the extensors. The second predictor turned out to be statistically significant (see Table 5). A decrease of the bilateral extensors’ imbalance by 8% decreased the THA surgery probability by 8%. The model explained almost 22% of the data variance (Nagelkerke R^2^ = 0.22).

**Figure 2:**
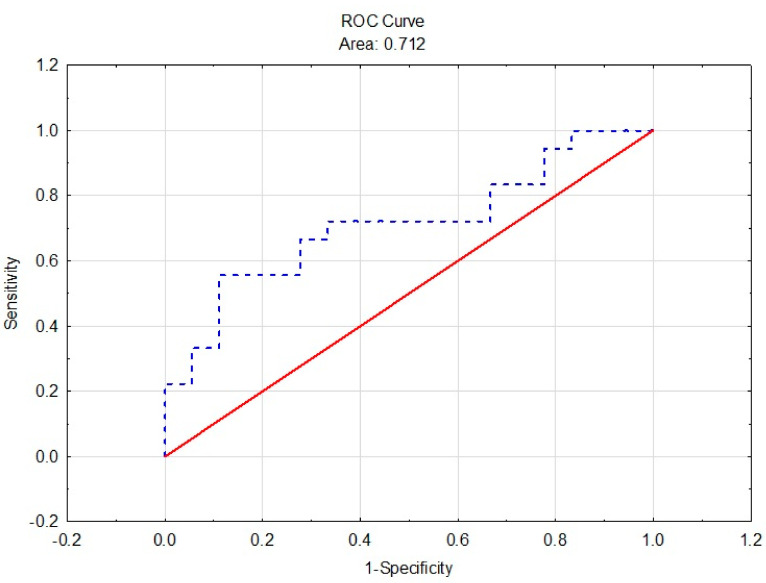
ROC curve of the bilateral extensor imbalance (% Ext).

The results of the analysis ROC curve shows that the % Ext of the bilateral imbalances has acceptable discrimination (AUC = 0.712 ± 0.087, 95% CL = 0.543–0.883) (see Figure 2). The ipsilateral imbalance between the extensors and the flexors in the CON and THA groups was used as the predictor in the second logistic model. The predictor turned out to be insignificant (see Table 6); hence, we did not perform a ROC analysis. The model explained about 2% of the data variance (Nagelkerke R^2^ = 0.02).

## 4. Discussion

Our study’s objective was to compare the strength of the knee joint muscle in the affected and unaffected lower extremities (i.e., bilateral and ipsilateral strength imbalances) in the preoperative period in patients indicated for THA due to OA with that of healthy elderly people in the CON group. We also aimed to assess whether imbalances may be used as predictors for THA surgery, i.e., as another clinical indicator.

It has been demonstrated that a better preoperative health status (e.g., greater physical function and strength) is a strong predictor of a good postoperative outcome following joint replacement [20,21]. The evidence suggests that preoperative rehabilitation programs proved significant improvements in the postoperative outcomes i.e., pain, function and length of hospital stay in patients undergoing THA [22]. We observed impairment of the knee extensors in the affected lower extremity as compared to the unaffected lower extremity in the THA group. Furthermore, in a comparison between the groups (THA and CON), we observed the impairment of the knee flexors in the affected and unaffected extremities in the THA group. We observed a significant bilateral strength imbalance of the knee extensors (% Def = 18.51%) in the THA group as compared to the CON group (% Def = 8.82%). A 10–15% bilateral strength imbalance between the lower extremities [23,24] is considered significant in the healthy population. Sufficient muscle strength in the quadriceps is crucial, because it has been shown to be related to patient-reported outcomes and physical functioning in THA patients, therefore indicating a strong predictive relationship with daily functional activities, e.g., walking and stair climbing [11].

On the other hand, in the THA group as compared to the CON group, we also observed the ipsilateral imbalances H/Q = 47.69% on the affected extremity and H/Q = 42.04% on the unaffected extremity. The referred H/Q ratio in the elderly population is 57.60–59.20% [25,26]. The referred low H/Q ratio in the THA group points to the weakening of the hamstring muscle strength in both extremities. Our findings are in agreement with previous studies reporting the weakening of the extensor muscle strength in the affected extremity [17,27] without significant differences between the affected and unaffected lower extremities in the flexor muscles [27]. Our research is also in line with another study [15] reporting the weakening of the extensor and flexor muscles in the affected lower extremities in THA patients as compared to the control group.

Regarding our second objective, i.e., the assessment of imbalances and their potential value as THA predictors, we found that the bilateral extensor imbalance may be used as a predictor for THA. The bilateral extensor imbalance was quite promising in terms of its diagnostic and prognostic values in clinical screening (Nagelkerke R^2^ = 0.22). A decrease of the bilateral extensors imbalance by 8% decreased the probability of THA by 8%.

Moreover, it has to be emphasized that there is poor agreement between frequent hip pain and radiographic osteoarthritis in the ipsilateral hip [28]. Most patients with frequent hip pain did not exhibit radiographic hip OA, and most patients with radiographic hip OA did not have recurring hip pain. On the other hand, MRI testing has shown that scanning can detect cartilage damage in those without radiographic knee osteoarthritis [29,30]. An MRI can also detect other abnormalities not seen on X-ray imagining, such as bone marrow lesions, synovitis and subtle osteophytes [29]. The problem with MRIs is that they are even more expensive than X-rays and less accessible. The evaluation of the imbalances seem to be a rather convenient and healthier way of testing the progression of OA, and it can be promising, because radiographic methods require patients to be exposed to radiation and should not be used too often.

## 5. Conclusions

In this study, we present the assessment of the maximal knee extensor and flexor strength and evaluate the bilateral and ipsilateral imbalances in thirty-six participants. Two groups were analyzed: patients that indicated THA due to hip OA and healthy elders. The purpose of our study was to compare the knee joint’s muscle imbalances in the affected and unaffected lower extremities in the preoperative period of patients facing THA and assess whether the muscle imbalances may be used as another clinical predictor for THA surgery, i.e., as another clinical indicator tested during symptom screening.

The most interesting result is that the evaluation of the bilateral extensor imbalance may be used as another clinical predictor for THA. However, this statement needs to be considered with caution. Although radiography screening, i.e., the most used method in hip OA progression, does not correlate fully with hip pain symptoms, we cannot claim that the imbalance itself is a sufficient predictor for THA surgery or can replace radiography.

This approach is promising, because if confirmed, bilateral strength imbalances can be used in patient screening as a clinical diagnostic tool. Isokinetic dynamometry is widely used by physiotherapists and is much more common in outpatient clinics of the orthopedic department than other more expensive diagnostic equipment (e.g., X-ray or MRI).

The strengths of this study have to be acknowledged. First of all, to the best of our knowledge, it is the very first study assessing whether muscle imbalances may be used as THA predictors.

We must also list the limitations of the study. The number of participants in our study was too low to draw decisive conclusions. Our findings, therefore, have to be confirmed in other studies. Perhaps a follow-up study or a study performed on a larger/another population would result in a better fit (since the model fitting relies heavily on the number of observations). The limitations of this study may serve as recommendations for future research.

## Figures and Tables

**Table 1:** Characteristics of the THA and CON groups.

Group	THA *n* = 18	CON *n* = 18	Min	Max	Percentiles			*U*	*p*	*d*	95% CI
					25th	50th	75th				
Age (yrs)	58.38 ± 5.30	64.64 ± 3.57	48.09	67.53	59.37	63.12	65.51	45.00	0.000	1.39	[0.66, 2.11]
Height (cm)	178.67 ± 6.58	175.81 ± 7.41	164.70	190.00	172.25	177.30	181.38	202.50	0.203	0.41	[−0.25, 1.07]
Weight (kg)	99.41 ± 17.81	95.54 ± 14.58	69.20	134.00	84.95	96.55	107.15	186.00	0.462	0.24	[−0.42, 0.89]

THA: patients indicated for Total Hip Arthroplasty, CON: Control Group—healthy elderly, U: nonparametric Mann–Whitney *U* test, *p*: *p*-value, d: Effect size index d (Cohen’s *d*) and CI: confidence interval.

**Table 2:** Evaluation of the bilateral strength imbalances in the THA and CON groups.

Group	Move	Lower Extremity	*n*	Percentiles Peak Torque (Nm)			*U*	*p*	*d*	95% CI
				25th	50th	75th				
THA	Ext	UNE	18	145.00	178.00	182.75	83.50	0.013	0.90	[0.22, 1.59]
		AFE	18	115.75	131.00	162.25				
THA	Flex	UNE	18	57.25	69.50	81.00	132.50	0.350	0.32	[−0.34, 0.97]
		AFE	18	53.75	64.00	79.00				
CON	Ext	SE	18	140.00	157.50	174.75	126.50	0.261	0.42	[−0.24, 1.08]
		WE	18	127.50	148.50	173.00				
CON	Flex	SE	18	73.00	89.50	98.75	112.50	0.017	0.51	[−0.16, 1.17]
		WE	18	68.00	76.50	87.25				

THA: patients indicated for Total Hip Arthroplasty, CON: Control Group—healthy elderly, UNE: unaffected extremity, AFE: affected extremity, SE: stronger extremity, WE: weaker extremity, Ext: extension, Flex: flexion, U: nonparametric Mann–Whitney *U* test, *p*: *p*-value, *d*: Cohen’s *d* and CI: confidence interval.

**Table 3:** Assessment of the extensor and flexor muscle strength imbalances between groups.

Group	Move	Lower Extremity	*n*	*U*	*p*	*d*	95% CI
THA CON	Ext	UNE	18	125.00	0.242	0.41	[−0.25, 1.08]
		SE	18				
THA CON	Flex	UNE	18	87.50	0.018	0.83	[0.15, 1.51]
		SE	18				
THA CON	Ext	AFE	18	138.50	0.457	0.21	[−0.45, 0.86]
		WE	18				
THA CON	Flex	AFE	18	92.50	0.028	0.71	[0.04, 1.38]
		WE	18				

THA: patients indicated for Total Hip Arthroplasty, CON: Control Group—healthy elderly, UNE: unaffected extremity, AFE: affected extremity, SE: stronger extremity, WE: weaker extremity, Ext: extension, Flex: flexion, U: nonparametric Mann–Whitney *U* test, *p*: *p*-value, *d*: Effect size index d (Cohen’s *d*) and CI: confidence interval.

**Table 4:** Comparison of the bilateral (% Def) and ipsilateral (H/Q) strength imbalances between the groups.

Muscle Imbalance	Group	Move	Lower Extremity	% Def; H/Q	*U*	*p*	*d*	95% CI
Bilateral	THA	Ext	UNE	18.51 ± 15.06	93.00	0.029	0.82	[0.14, 1.50]
			AFE					
Bilateral	CON	Ext	SE	8.82 ± 7.28				
			WE					
Bilateral	THA	Flex	UNE	14.40 ± 10.25	136.00	0.411	0.29	[−0.37, 0.94]
			AFE					
Bilateral	CON	Flex	SE	11.76 ± 8.12				
			WE					
Ipsilateral	THA	Flex	AFE	47.69 ± 9.69	95.00	0.034	0.72	[0.05, 1.40]
		Ext	AFE					
Ipsilateral	CON	Flex	WE	54.28 ± 8.55				
		Ext	WE					
Ipsilateral	THA	Flex	UNE	42.04 ± 7.43	34.00	0.000	1.75	[0.98, 2.52]
		Ext	UNE					
Ipsilateral	CON	Flex	SE	55.71 ± 8.16				
		Ext	SE					

THA: patients indicated for Total Hip Arthroplasty, CON: Control Group—healthy elderly, UNE: unaffected extremity, AFE: affected extremity, SE: stronger extremity, WE: weaker extremity, Ext: extension, Flex: flexion, U: nonparametric Mann–Whitney *U* test, *p*: *p*-value, *d*: Effect size index d (Cohen’s *d*) and CI: confidence interval.

**Table 5:** Logistic regression model results with bilateral imbalances as predictors.

Effect	*df*	β	S.E.	Wald	*p*	Odds Ratio	95% CI for Odds Ratio	
							Lower	Upper
Intercept	1	1.223	0.757	2.609	0.106			
% Ext	1	−0.083	0.042	3.898	0.048	0.920	0.847	0.999
% Flex	1	−0.013	0.044	0.085	0.771	0.987	0.906	1.076

% Ext: bilateral strength imbalances (percent) between extensors in unaffected and affected (THA group) and stronger and weaker (CON group) lower extremities. % Flex: bilateral strength imbalances between flexors in unaffected and affected (THA group) and stronger and weaker (CON group) extremities.

**Table 6:** Logistic regression model results with ipsilateral imbalances as a predictor.

Effect	*df*	β	S.E.	Wald	*p*	Odds Ratio	95% CI for Odds Ratio	
							Lower	Upper
Intercept	1	0.449	1.411	0.101	0.750			
% diff ipsilateral	1	−0.031	0.029	1.212	0.271	1.032	0.976	1.091

% diff (ipsilateral): ipsilateral difference (percent) between the flexors and extensors in their respective lower extremities.

## Data Availability

The data presented in this study are available upon request from the corresponding author. The data are not publicly available due to ethical restrictions.
